# Probiotics, Prebiotics, Synbiotics, Postbiotics, and Paraprobiotics—New Perspectives on Functional Foods and Nutraceuticals

**DOI:** 10.3390/foods14152613

**Published:** 2025-07-25

**Authors:** Zhongyuan Li, Xuegang Luo

**Affiliations:** 1 Key Laboratory of Industrial Fermentation Microbiology, Ministry of Education, Tianjin 300457, China; lizhongyuan@tust.edu.cn; 2 Tianjin Key Laboratory of Industrial Microbiology, College of Biotechnology, Tianjin University of Science and Technology, Tianjin 300457, China

## 1. Introduction

Probiotics and their derivatives (including prebiotics, synbiotics, postbiotics, and paraprobiotics) have been extensively proven to regulate the gut microbiota balance and promote host health [1]. Their applications are rapidly expanding into multiple fields such as food, healthcare, and functional products. In the food industry, probiotics serve not only as core functional components in fermented dairy products (e.g., yogurt, cheese), but are also innovatively incorporated into plant-based beverages, baked goods, and snack foods to meet the consumer demand for “gut-friendly” healthy foods [2]. In the medical and health sector, probiotic formulations are already used as adjunctive therapies for metabolic disorders, intestinal inflammation, and immune regulation. Specific metabolites from certain strains even demonstrate the potential to replace conventional pharmaceuticals [3]. Thus, this Special Issue gathered outstanding scientific publications from related fields to discuss health effects, mechanisms, and the development of research approaches.

## 2. An Overview of the Published Articles

There are six research articles and two reviews published in this Special Issue. Four research articles studied the health functions of probiotics, such as their anti-allergic effects, anti-obesity effects, and ability to reduce hyperuricaemia. Xu et al. evaluated the anti-allergic effects of Lactobacillus kefiranofaciens ZW3 (ZW3) in ovalbumin (OVA)-induced allergic mice [4]. The results suggest that the body weight and thymus index returned to normal levels; the allergy scores, serum OVA-sIgE, IL-4, IL-5, and IL-10 expression decreased; and IFN-γ and IL-2 increased significantly in the ZW3 group compared with the allergy group. Furthermore, ZW3 decreased Muribaculaceae and Ruminococcaceae abundance and increased Lachnospiraceae abundance in the intestinal flora. In summary, ZW3 induced anti-allergic effects by increasing the number of Th1 cytokines and decreasing that of Th2 cytokines, which can remarkably ameliorate the symptoms of an ovalbumin-induced food allergy. In another article, a novel probiotic yeast, *Kluyveromyces lactis* JSA 18, isolated from yak milk, demonstrated significant anti-obesity effects in mice fed a high-fat diet (HFD) [5]. *K. lactis* JSA 18 reduced weight gain, liver/fat indexes, hyperlipidemia, serum triglycerides, and liver enzymes (ALT/AST). It also suppressed inflammatory cytokines (TNF-α and IL-1β) and enhanced lipid metabolism by downregulating key genes (ACC1, PPAR-γ, SREBP-1, and Fasn). Ren et al. isolated a novel probiotic, *Pediococcus acidilactici* GQ01, from fermented wolfberry [6]. *Pediococcus acidilactici* GQ01 and its heat-killed G1PB postbiotic both effectively treated hyperuricemia (HUA) in mice. They reduced the uric acid, creatinine, and urea nitrogen in blood. GQ01 primarily inhibited ADA activity, while G1PB inhibited XOD. They alleviated liver/kidney damage, upregulated kidney ABCG2 and liver XOD, and downregulated kidney URAT1/GLUT9 transporters, promoting uric acid excretion. Both restored the healthy gut microbiota structure and increased the amount of beneficial short-chain fatty acids (acetic, propionic, and butyric acid). This demonstrates GQ01 and G1PB’s potential as functional food/drug materials for HUA with minimal side effects. Gamma-aminobutyric acid (GABA) is a non-protein amino acid crucial for various physiological functions, primarily acting as an inhibitory neurotransmitter in mammals. Ten Lactiplantibacillus plantarum strains with a high Gamma-aminobutyric acid (GABA)-producing ability were screened from Chinese Paocai [7]. Based on the one-factor-at-a-time strategy and the response surface methodology (RSM) analysis, *Lp. plantarum* FRT7 showed the highest GABA yield of 1158.6 ± 21.22 mg/L, meaning that it has the potential to be a health-beneficial probiotic with commercial capabilities.

Encapsulation techniques play a crucial role in enhancing the stability and viability of probiotics in functional foods. Chusak et al. found that calcium alginate encapsulation combined with hydrocolloids significantly boosted Lactobacillus rhamnosus GG viability and the total phenolic content (TPC) in fermented black goji berry beads versus alginate alone [8]. Calcium alginate–gelatin beads showed the highest level of probiotic survival after simulated digestion. Calcium alginate–carrageenan beads best preserved viability when co-digested with milk. Co-ingestion with any milk type enhanced TPC retention across all bead types, as the milk macronutrients protected polyphenols during digestion. This approach enhances probiotic and polyphenol stability in non-dairy beverages.

In Vitro digestion models offer a rapid, cost-effective assessment of food digestion and health impacts. While edible polysaccharides’ role in gut health has gained attention, systematic reviews on microbial polysaccharides (e.g., xanthan gum, gellan gum) in these models remain scarce. Wang et al. reviews the limitations of static/dynamic digestion experiments and introduces key models [9]. They focus on the degradability of microbial polysaccharides/oligosaccharides and their effects on intestinal health. The work aims to advance the understanding of these compounds’ applications, particularly in probiotic delivery, immobilization, and probiotic potential, providing deeper insights into functional food development. In addition, Li et al. applied an in vitro fermentation study to the effects of five human milk oligosaccharides (HMOs) on the gut microbiota and the short-chain fatty acid (SCFA) metabolites of infants aged 0–6 months [10]. The results indicated that the initial microbial community composition significantly influenced the fermentation outcomes, and that in vitro fermentation maintained dominant bacterial species within the gut, but also affected their abundance and distribution. In addition, most test Bifidobacterium species effectively utilized HMOs/their degradation products and produced different levels of acetate. These findings suggest the potential applications of HMOs in developing food products targeting infant microbiomes.

Rising interest in paraprobiotics and postbiotics as health agents has driven scientific efforts to understand their bioactivity and production. The second review article employs a bibliometric analysis of the Web of Science literature to map the evolution of the research and identify bottlenecks [11]. The results show that current studies primarily focus on bioactivity. However, significant research gaps persist in the production methods and food interactions for functional food development. Crucially, further validation is needed to substantiate the bioactivity claims, especially for functional food applications.

## 3. Conclusions

In summary, this Special Issue “Probiotics, Prebiotics, Synbiotics, Postbiotics, and Paraprobiotics—New Perspectives on Functional Foods and Nutraceuticals” demonstrates the significant interest in exploring the health functions of probiotics and their derivatives, as well as in developing research approaches that contribute to enhancing the efficiency and depth of research.

## References

[1] Rudnyckyj S., Thomsen M.H. (2025). Probiotics and Postbiotics Derived from Saline/Marine Plant-Based Feedstocks. Probiotics Antimicrob. Proteins.

[2] Moshfeghinia R., Nemati H., Ebrahimi A., Shekouh D., Karami S., Eraghi M.M., Mohagheghzadeh H., Hunter J., Pasalar M.J. (2025). The Impact of Probiotics, Prebiotics, and Synbiotics on Depression and Anxiety Symptoms of Patients with Depression: A Systematic Review and Meta-analysis. Psychiatr. Res..

[3] Nataraj B.H., Ali S.A., Behare P.V., Yadav H. (2020). Postbiotics-Parabiotics: The New Horizons in Microbial Biotherapy and functional Foods. Microb. Cell Fact..

[4] Xu H., Duan X., Wang Y., Geng W. (2025). Amelioration Effect of *Lactobacillus kefiranofaciens* ZW3 on Ovalbumin-Induced Allergic Symptoms in BALB/c Mice. Foods.

[5] Hong Y., Song G., Feng X., Niu J., Wang L., Yang C., Luo X., Zhou S., Ma W. (2024). The Probiotic Kluyveromyces lactis JSA 18 Alleviates Obesity and Hyperlipidemia in High-Fat Diet C57BL/6J Mice. Foods.

[6] Ren L., Wang S., Liu S., Prasanthi H.A.C., Li Y., Cao J., Zhong F., Guo L., Lu F., Luo X. (2024). Postbiotic of Pediococcus acidilactici GQ01, a Novel Probiotic Strain Isolated from Natural Fermented Wolfberry, Attenuates Hyperuricaemia in Mice through Modulating Uric Acid Metabolism and Gut Microbiota. Foods.

[7] Cai H., Li X., Li D., Liu W., Han Y., Xu X., Yang P., Meng K. (2023). Optimization of Gamma-Aminobutyric Acid Production by Lactiplantibacillus plantarum FRT7 from Chinese Paocai. Foods.

[8] Chusak C., Balmori V., Kamonsuwan K., Suklaew P., Adisakwattana S. (2025). Enhancing Viability of Lactobacillus rhamnosus GG and Total Polyphenol Content in Fermented Black Goji Berry Beverage Through Calcium–Alginate Encapsulation with Hydrocolloids. Foods.

[9] Wang Y., Zhu S., Zhang T., Gao M., Zhan X. (2024). New Horizons in Probiotics: Unraveling the Potential of Edible Microbial Polysaccharides through In Vitro Digestion Models. Foods.

[10] Li M., Lu H., Xue Y., Ning Y., Yuan Q., Li H., He Y., Jia X., Wang S. (2024). An In Vitro Colonic Fermentation Study of the Effects of Human Milk Oligosaccharides on Gut Microbiota and Short-Chain Fatty Acid Production in Infants Aged 0–6 Months. Foods.

[11] Monteiro S.S., Schnorr C.E., Pasquali M.A.d.B. (2023). Paraprobiotics and Postbiotics—Current State of Scientific Research and Future Trends toward the Development of Functional Foods. Foods.

